# Environmental Impact of Concrete Slab Made of Recycled Aggregate Concrete Based on Limit States of Load-Bearing Capacity and Serviceability—LCA Case Study

**DOI:** 10.3390/ma16020616

**Published:** 2023-01-09

**Authors:** Tereza Pavlů, Jan Pešta, Tomáš Vlach, Kristina Fořtová

**Affiliations:** University Centre for Energy Efficient Buildings, Czech Technical University in Prague, Třinecká 1024, 27343 Bustehrad, Czech Republic

**Keywords:** fine recycled aggregate, recycled concrete aggregate, recycled masonry, aggregate, recycled aggregate concrete, concrete slab, LCA, limit states of load-bearing capacity, limit states of serviceability

## Abstract

In the case of concrete sustainability, two main ways are generally discussed: (1) the reduction of natural raw materials and (2) the reduction of emissions related to concrete production. Following the second point, there have not yet been reported clear results. This problem is not given enough attention in present publications. This study brings a general view of this issue and a basic comparison with common concrete and traditional reinforcement. This case study deals with the life cycle analysis of a concrete slab made of recycled aggregate concrete with a fine recycled aggregate. The concrete slab was designed according to the limit states of load-bearing capacity and serviceability, which is based on the experimental verification of recycled aggregate concrete properties. Two different reinforcements are compared: (1) ordinary reinforcement by steel bars and (2) glass fibers. Furthermore, scenarios vary due to the slab thickness and reinforcement percentage. The results show the positive environmental impact of replacing natural sand with a fine recycled aggregate. The reduction of climate change potential can be almost 40% in some cases.

## 1. Introduction

Concrete, the most used construction material, is generally known as the largest consumer of natural resources in the construction industry. For this reason, one of the main possibilities to reduce the negative environmental impact of the building sector is to find a more sustainable solution in concrete production. It could be said that the clear way is to replace the natural aggregate (NA), which is approximately 70% of the largest component of concrete, with a recycled one (RA) [1]. However, from the CO_2_ emissions point of view, the aggregate is a small emitter in whole concrete production (~15%) [2] compared to cement production and transportation. Moreover, the environmental impact of an RCA is typically influenced by its transportation scenario [3,4,5,6,7]. Furthermore, it is generally known that the use of a RA is usually redeemed by the decline of mechanical properties and the durability of recycled aggregate concrete (RAC) in comparison with conventional concrete (NAC). This decline is usually compensated by adding cement, which causes a worse environmental profile, or supplementary cementitious material (SCM) [8]. Furthermore, the partial replacement of a NA up to 30% of a coarse fraction is without a decline of properties; however, it has been presented that only the partial replacement of a NA has no significant impact on the decrease of greenhouse emissions [9].

The majority of the published studies dealing with the life cycle assessment (LCA) of RAC investigate the impact of coarse fraction replacement [4,5,6,7,10,11,12,13,14,15,16]. Thus, due to the research into concretes with a coarse RA being very advanced, the results have been verified multiple times and there are probably no longer any discrepancies in its use. On the contrary, only a few LCA studies have been published focused on the structural use of fine RAs (fRA) [17]. The probable reason is that from the LCA point of view, it has been found that the full replacement of natural sand reduces CO_2_ emissions by only up to 2% [18]. Moreover, the utilization of a fRA is related to many uncertainties, such as hardly measurable water absorption, which leads to the unknown effective water–cement ratio [18,19]. Furthermore, the particle shape, higher amount of fines, the threat of impurities content, etc. is mostly influenced by the recycling procedure [18]. Furthermore, the treatment technology causes differences in environmental footprints, although the aim is to increase the fRA quality and optimize the mixture [20].

On the other hand, it is necessary to know that the sand supplies are not infinite and the extraction of sand induces much environmental damage worldwide, including the erosion and damage of landscapes.

Furthermore, the LCA is strongly affected by the functional unit (FU), system boundaries, allocation, LCI, and others. Historically, only the RA and NA have been compared [21], and then the 1 cubic meter of RAC has been environmentally assessed [6,22]. Gradually, other aspects of the use of a RA in concrete have been added such as mechanical properties [7,23,24,25], durability, etc. [11,15,26]. Finally, the structural applications of RAC started to be environmentally analyzed [22,27,28,29]. From the point of view of the functional comparing of structures by the LCA method, two main principles have been defined [30]. In Principle I, which is primarily applied, the FU is related to mechanical properties, the volume of the structural element is constant, and the service life of the structural element varies according to the durability of the material [31,32,33]. In Principle II, the FU depends on mechanical properties, the service life of the structural elements stays constant, and the volume of the structural element varies according to the durability of the material such as freeze–thaw, carbonation, and chloride resistance.

The main aim of this study is to analyze the environmental impact of concrete slabs made of RAC where the natural sand (fNA) is fully replaced by the fRA (fine recycled masonry aggregate fRMA and fine recycled concrete aggregate fRCA). The design of the slab was based on the limit states of load-bearing capacity (LBC) and serviceability (SA). The design of the structural elements is established according to the experimentally verified properties of concrete. The LCA method of evaluation of the environmental impact was used for this case study. The novelty of this study is a comprehensive approach to the LCA case study of the concrete structural elements with the full replacement of sand in the mixture, various concrete mixtures, reinforcement types, and design approach.

## 2. Materials and Methods

**Figure 1:**
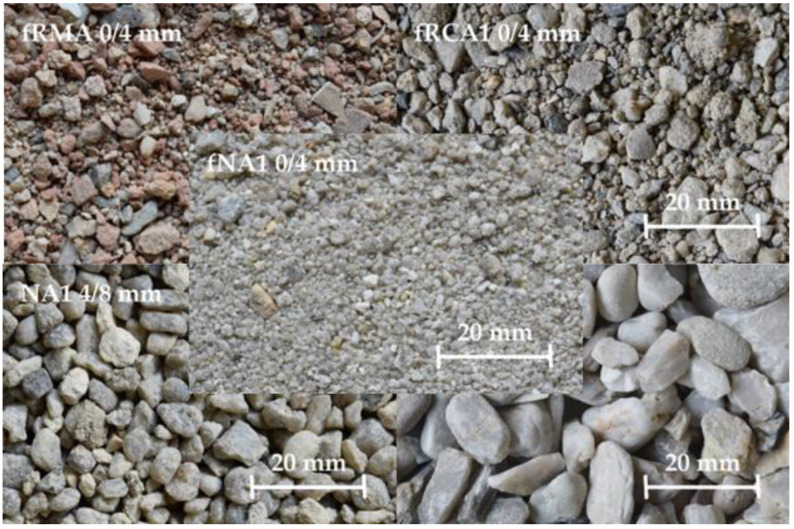
NA and RA used in concrete mixtures.

In total, six concrete mixtures were experimentally verified for the possible replacement of natural sand by an fRA. The concrete mixtures contained two types of fRA (fraction 0–4 mm), coarse NA (fraction 4–16 mm) (see Figure 1), two amounts of cement, and various water–cement ratios (I and II). The mechanical and durability properties related to the structural use were experimentally verified, and the result values were considered for the design of the concrete slab. Various reinforcements of the concrete slab were considered: traditional steel reinforcement bars (S) and glass fiber reinforcement (G). Furthermore, two scenarios were compared: Scenario 1, where the thickness of the slab varies, and Scenario 2, where the reinforcement level varies, both according to the mechanical properties of concrete with the consideration of durability properties. Moreover, the two limit states were considered: (1) load-bearing capacity and (2) serviceability. The LCA method was used for environmental assessment performed by the GaBi software v2022.2. The functional unit was considered 1 square meter of a concrete slab. The cradle-to-grave system boundaries were established.

### 2.1. Fine Recycled Aggregate

The two types of fRA were used as a replacement for natural sand in the concrete mixture. The fRCA is produced by crushing waste concrete from CDW [34,35,36,37] and the fRMA originated from waste masonry [38,39,40,41]. Both types of aggregate originate from a recycling center in the Czech Republic. As described in previous studies [18,19], the measurement method of fRA’s density and water absorption has not yet been established; the method defined in the Standard ČSN EN 1097-6 was used for the determination (see Table 1).

### 2.2. Recycled Aggregate Concrete

The six concrete mixtures were produced for experimental examination of the mechanical properties and durability. Mixture I contained 260 kg/m^3^ ordinary Portland cement (OPC) CEM I 42.5 R and the predicted effective water–cement ratio was 0.65, and mixture II contained 300 kg/m^3^ cement CEM I 42.5 R and the predicted effective water–cement ratio was 0.55. The target effective water–cement ratio was determined from the water absorption of the fRA and predicted effectiveness of sorption capacity during mixing. The Bolomey particle size distribution curve was used for optimizing the skeleton of the concrete mixtures. The two-stage mixing approach [42] was used for concrete mixing. The mixture proportions are shown in Table 2.

### 2.3. Recycled Aggregate Concrete Properties

The physical and mechanical properties and frost and carbonation resistance, the properties relevant to the structural utilization, were experimentally verified. The results of the evaluation were used for the structural design. The compressive strength was tested on cubes 150 × 150 × 150 mm^3^, and the modulus of elasticity and flexural strength were tested on beams 100 × 100 × 400 mm^3^, all at age 28 days by Controls MCC8 50-C8422/M (Controls Group, Milan, Italy) according to the relevant standards (ČSN EN 12390-3, ČSN EN 12390-5, and ČSN EN 12390-13). The carbonation resistance (ČSN EN 12390-12) was tested on beam samples 100 × 100 × 400 mm^3^ at age 28 days by CO2CELL (MMM group) and freeze–thaw resistance was tested according to ČSN 73 1322 on beam samples 100 × 100 × 400 mm^3^ at age 28 days for 100 freezing–thawing cycles by KD20 (Ecofrost, Olomouc, Czech Republic).

In previous studies, it has been presented that the compressive strength is significantly influenced by the fRA until 30% of the substitution level; however, in the case of modulus of elasticity, the decline can be observed already at lower replacement ratios [18]. For this reason, in this case study, two limit states were considered, the load-bearing limit state which is related to the compressive strength, and the serviceability depending on the modulus of elasticity. The experimentally verified data was used for the designing of the concrete slab. The used results are shown in Table 3. The concrete cover of steel reinforcement was considered based on the results of depth of carbonation (see Table 4).

### 2.4. Environmental Assessment

To analyze the potential environmental impacts of product systems, the LCA method was used. According to ISO 14040:2006 [43], LCA was performed in four main phases: the goal and scope definition, life cycle inventory (LCI), life cycle impact assessment (LCIA), and life cycle interpretation [44].

#### 2.4.1. Goal and Scope Definition, Functional Unit, and System Boundaries

The goal of this study was to compare six concrete mixtures used in concrete slabs containing glass or steel reinforcements, which were designed to fulfill requirements for the limit states of load-bearing capacity and serviceability. 

In this study, the function of compared concrete slabs differ based on their limit states. Therefore, designs related to the limit states of load-bearing capacity and serviceability are compared separately.

The product systems were compared in the scope of system boundaries, which can be described as cradle-to-grave. Thus, system boundaries cover the production of raw materials (including recycling processes for the production of recycled aggregates) and their transport, preparation of the concrete mixture, production of the concrete slab, deconstruction, and its assumed end of life (EoL), which consists of deconstruction, transport for disposal, and landfilling as the most common process for the disposal of concrete in the Czech Republic.

#### 2.4.2. Life Cycle Inventory (LCI)

The referential flows of concrete mixtures for each slab are summarized in Table 5 and Table 6, where the calculated volume of concrete is described together with the amount of reinforcement.

The GaBi 9 software was used to conduct the data and model the product system. The production of recycled aggregates was modeled based on specific data from the producer and other processes were modeled using generic data from the GaBi 9 database [45]. For the basic scenario, distances for the transport of resources and waste were assumed to be 50 km.

#### 2.4.3. Life Cycle Impact Assessment, Normalization, and Weighing

The potential environmental impacts related to the considered product systems were calculated according to the environmental footprint (EF), version 3.0 characterization method. Using normalization and weighting of these results, the overall impact of each concrete slab was calculated. Normalized results were estimated by relating the results of the environmental indicators to the global impact using the factors according to EF 3.0 personal equivalents provided in the GaBi software v2022.2. To consider the specific value of each impact category, the weighing factors according to EF 3.0 were applied using the GaBi software. 

### 2.5. Design of Structural Elements

The results of the calculations provide basic data on material consumption for the next step to evaluate the LCA and make an overall evaluation. The design and calculation of the limit states of load-bearing capacity and serviceability were performed according to the Eurocode 2, specifically the ČSN EN 1992-1 standard. A typical ceiling panel with a span of 6 m was chosen to calculate and compare the variants. The panel is solid without cavities and lightening, and a common meter of panel width is considered. 

The input parameter for the calculations was the experimentally determined compressive strength of concrete for different formulas. Furthermore, the static modulus of elasticity of concrete was entered into the calculation, which was also determined experimentally. The E modulus was included for the serviceability limit state. The input parameters for the reinforcement were the mechanical parameters for traditional steel reinforcement, the tensile strength *f_yk_* 500 MPa, and the modulus of elasticity *E_s_* 200 GPa. For the AR-glass FRP reinforcement data from the technical data sheet reference, the real tensile strength *f_yk,real_* 1050 MPa and the real modulus of elasticity *E_s,real_* 50 GPa were used.

The constant floor live load *q* category A was considered, which means 1.50 kN/m^2^, and also moveable partitions 1.2 kN/m^2^. Additionally, floor self-weight *g1* was a constant 2.05 kN/m^2^ calculated from the self-weight of the typical selected floor composition. The self-weight of the reinforced concrete panel *g2* was calculated based on its designed thickness. The sum of these loads is the continuous load of the panel. With the considered width of 1.0 m and the simply supported panel, it is then easy to calculate the bending moment.

## 3. Results and Discussion

### 3.1. Results

Table 3 presents the results of the optimized calculation focused on the limit states of load-bearing capacity. The design was performed according to the ČSN EN 1992-1 standard. The basic thickness of the concrete panel was always optimized for NAC concrete according to all conditions of the load-bearing capacity with an ordinary amount of reinforcement. It means the ordinary diameter and spacing of reinforcement for the considered panel span. Concrete cover *c* of the reinforcement was considered as a constant regarding the durability of traditional steel reinforcement. A thickness of 20 mm for reference concrete from a natural aggregate and 35 mm for concrete with a recycled aggregate of concrete cover was considered, based on the results of carbonation resistance according to the ČSN EN 12390-12 standard. For the AR-glass FRP reinforcement, there is no need to propose the reinforcement concrete cover regarding durability, but only regarding the aggregate size and interaction conditions of the reinforcement and concrete, allowing full reinforcement activation. A constant value of 15 mm was selected as the concrete cover of AR-glass FRP reinforcement.

The calculations for the first group in Table 3, ‘*Steel reinforcement, thickness modification*’, for the limit state of load-bearing capacity includes traditional steel reinforcement and adjustment of the concrete thickness to comply with the conditions of the load-bearing capacity for different concrete mixtures and other sub-conditions of the calculation. The amount of reinforcement in the cross-sectional area was constant for this case; the reinforcement diameter was considered as 10 mm with an axial distance of 100 mm. The thickness of the panel was changed according to the measured strength of the concrete mixture.

The next group in Table 3, ‘*GFRP reinforcement, thickness modification*’, for the limit state of load-bearing capacity includes AR-glass FRP reinforcement and adjustment of the concrete thickness. The amount of reinforcement in the cross-sectional area was also constant for this case; the reinforcement diameter was considered as 6 mm thanks to its better tensile strength with an axial distance of 75 mm. The effect of the lower concrete cover was also positive. The thickness of the panel was very slightly changed to achieve the necessary load-bearing capacity. It was also possible to notice a change in the thickness of the panel. This is due to the condition of restricting the position of the neutral axis of the panel in bending.

The group ‘*Steel reinforcement*, *reinforcement modification*’ in Table 3 for the limit state of load-bearing capacity presents results with steel reinforcement and the modification of the amount of reinforcement in the cross-sectional area. The diameter of the reinforcement bars was constant and the axis distance was modified to reach the required amount of reinforcement. Changes in the amount of reinforcement were insignificant.

The group ‘*GFRP reinforcement*, *reinforcement modification*’ in Table 3 for the limit state of load-bearing capacity presents the modification of the amount of reinforcement in the cross-sectional area with AR-glass FRP reinforcement. The diameter of the reinforcement bars was constant and the axis distance was modified to reach the required amount of reinforcement. Changes in the amount of reinforcement were seriously insignificant. It is also possible to notice one change in the thickness of the panel. This is due to the condition of restricting the position of the neutral axis of the panel in bending.

Next, Table 4 presents the results of the optimized calculation focused on the limit state of serviceability. The design was performed according to the ČSN EN 1992-1 standard. The decisive parameter is the panel deflection in the worst point—the middle of the panel span. The deflection calculation is the sum of the deflection of the long-term load, the short-term load, and the shrinkage. All these parameters were included in the calculation. The calculation of the limit state of serviceability was included because it is usually decisive in the construction design of horizontal structure elements, and this study presents two materials and their mutual combinations that can negatively affect the result due to the low elastic modulus. It is concrete with recycled aggregate and AR-glass FRP reinforcement. 

The basic thickness of the concrete panel was optimized for NAC according to the conditions of serviceability with a limited deflection in the middle of the span of the panel at 24 mm. In addition, conditions of the limit state of load-bearing capacity were of course satisfied, with an ordinary amount of reinforcement in the first step for NAC and steel reinforcement. It means that an ordinary diameter and spacing of reinforcements for the considered panel spanning concrete cover *c* of the reinforcement was considered, as in the previous case of the design for the limit state of load-bearing capacity. Then, the thickness of the panel or the reinforcement area was modified for different concrete classes with experimentally determined parameters and steel and AR-glass FRP reinforcement. Results were used in the next step to evaluate LCA.

The first group of calculations labeled ‘*Steel reinforcement, thickness modification*’ in Table 4 for the limit state of serviceability includes traditional steel reinforcement and adjustment of the concrete thickness to comply with the conditions of limiting the load-bearing capacity for different concrete mixtures and other sub-conditions of the calculation. The decisive condition of the proposal is the limited deflection in the middle of the span. The amount of reinforcement in the cross-sectional area was constant for this case; the reinforcement diameter was considered as 10 mm with an axial distance of 100 mm, just like in the case of the limit state of load-bearing capacity. The thickness of the panel was changed according to the measured strength of the concrete mixture and is higher in comparison with the same group in Table 3 for the limit state of load-bearing capacity.

The next group ‘*GFRP reinforcement, thickness modification*’ of Table 4 for the limit state of serviceability includes AR-glass FRP reinforcement and adjustment of the concrete thickness. The amount of reinforcement in the cross-sectional area was also constant for this case; the reinforcement diameter was considered as 6 mm thanks to its better tensile strength with an axial distance of 75 mm, just like in the case of the limit state of load-bearing capacity. The decisive condition of the proposal was clearly the limited deflection in the middle of the span. The thickness of the panel was rapidly changed to achieve the necessarily limited deflection. The negative effect of the low modulus of elasticity of both concrete and, especially, composite reinforcement is visible.

The group labeled ‘*Steel reinforcement*, *reinforcement. Modification*’ in Table 4 for the limit state of serviceability presents results with steel reinforcement and the modification of the amount of reinforcement in the cross-sectional area. The diameter of the reinforcement bars was higher, it was 12 mm, and the axis distance was also modified to reach the required amount of reinforcement. Changes in the amount of reinforcement were insignificant. Further, for this group, the decisive condition of the proposal was clearly the limited deflection in the middle of the span. The thickness of the panel had to be changed to 300 mm due to the condition of restricting the position of the neutral axis of the panel when bending and the minimal required amount of reinforcement.

The group labeled ‘*GFRP reinforcement*, *reinforcement modification*’ in Table 4 for the limit state of serviceability presents the modification of the amount of reinforcement in the cross-sectional area with AR-glass FRP reinforcement. The diameter of the reinforcement bars was constant at 10 mm and the axis distance was modified to reach the required amount of reinforcement. Changes in the amount of reinforcement were quite significant. It was also possible to notice changes in the thickness of the panel. This is due to the condition of restricting the position of the neutral axis of the panel when bending and the minimal required amount of reinforcement. The negative effect of the low modulus of elasticity of both the concrete and, especially, composite reinforcement is visible, as in the previous case. A higher amount of reinforcement does not have such a great effect on the overall consumption of material and thickness of the concrete panel. From the point of view of the proposal to assess the limit state of serviceability, it can therefore be stated that AR-glass FRP reinforcement seems unsuitable as a reinforcement of horizontal structural elements due to the low stiffness and low modulus of elasticity.

### 3.2. Discussion

**Figure 2:**
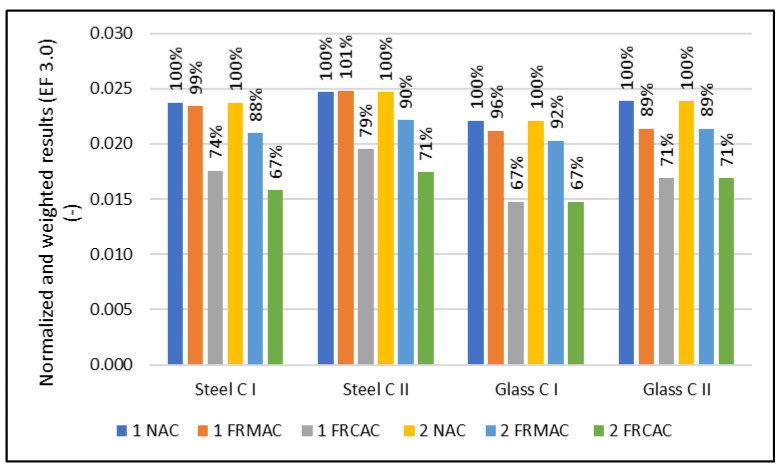
Normalized and weighted results for load-bearing capacity-oriented design scenarios (according to EF 3.0).

**Figure 3:**
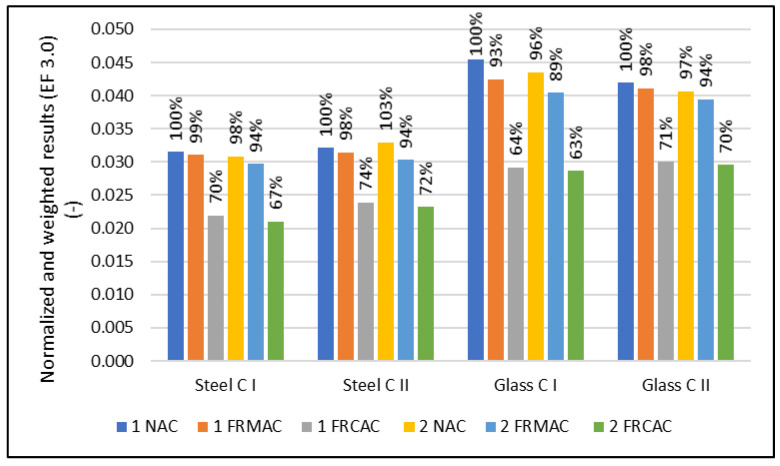
Normalized and weighted results for serviceability-oriented design scenarios (according to EF 3.0).

Previously published studies have reported that the use of an RA in concrete cannot lead to significant environmental benefits, which have been mainly limited to the reduction of landfill areas and raw materials extraction [46]. The probable reason is that although the aggregate represents approximately 70% of the concrete volume, the environmental impact of the production of cement and transportation is too essential, and so the savings from a natural aggregate cannot compete. The majority of studies have also shown the high sensitivity of results related to transportation [1]. Only a few studies have been published (around 19% of all [8]) dealing with the environmental assessment of RAC, where the fRA is considered as a possible substitution for natural sand [23,24,27,28,47,48,49,50,51,52,53,54]. In most investigations, it has been reported that the decline of mechanical properties and complications caused by the unknown water–cement ratio (which is related to higher porosity and water absorption) is too essential in comparison with the insignificant reduction of the environmental footprint, mostly related to the reduction of erosion and landscape damage. For this reason, it was argued by Kurda et al. [54], Dezhampanah et al. [48], and Roh et al. [47] that the use of an fRA more essentially influenced the compressive strength of RAC, whereas the environmental impacts are not affected. On the contrary, the studies by Evangelista and De Brito [17] and Yang et al. [53] quantified the reductions of the environmental footprint for mixtures with the full replacement of natural sand by an fRA with the same mix design. It obtained a reduction of 27% and 6.8%, respectively. However, the inconsistency of the compressive strength was not considered in both cases. Moreover, Evangelista and de Brito [17] observed that the environmental impact in the impact categories ADP, GWP, ODP, AP, EP, and POCP decreased by 6–8% when 30% of fRA was used and 19–23% for the full replacement of fine NA. In this case, the 1 m^2^ of a solid concrete slab had a thickness of 0.25 m with a constant reinforcement ratio. On the contrary, in this LCA case study, the environmental impact of the reinforcement concrete slab, where the decline of the properties is compensated by the additional slab height or reinforcement level, is analyzed. Although the decline of properties, especially the modulus of elasticity, caused an increase in the slab height or reinforcement level, the normalized and weighted results for concrete show the positive environmental impact of replacement fNA by an fRA (see Table 7 and Table 8, Figure 2 and Figure 3). This does not correspond with the majority of previously published studies, where the decrease of properties and low reduction of environmental impact has been found [47,48,54]. The divergent results in comparison with previous studies is an insignificant decline of mechanical properties when the natural sand is replaced by the fRA. Furthermore, the production of the recycled aggregate consumes less energy, can be used for demolition for new structures without the necessity of transportation, and, furthermore, brings a positive impact related to the recycling of steel bars which is usually contained to concrete waste.

The results of this case study slightly correspond with the study of Evangelista and de Brito [17], where a similar structural element was chosen; however, in this study, the fine recycled aggregate concrete was considered without coarse NA and furthermore, the reinforcement by steel bars and the thickness of the slab is considered as constant, without taking into account the decline of mechanical properties. For these reasons, the results of both studies are not comparable. 

Generally, the most positive influence can be observed for concrete slabs containing an fRCA, due to the benefit of the steel recycling of parent reinforcement concrete [28]. However, in comparison with fRMAC, the fRCAC mixtures reached a better mechanical performance, which led to a lower thickness of slab or lower reinforcement level, respectively. Moreover, the results show a better environmental profile of concrete slabs with a constant thickness and various reinforcements (1 NAC, 1 FRMAC, and 1 FRCAC) in comparison with the design optimizing the thickness instead of reinforcement (2 NAC, 2 FRMAC, and 2 FRCAC). This implies that the optimal way is to compensate for the decline of the compressive strength of RAC with a higher reinforcement level and then use more concrete, which increases the cement content. In a comparison of the reinforcement of steel bars and glass fibers, similar results can be observed. Nevertheless, in the assessment of cement, steel, and glass reinforcement, it must be noted that the production of 1 kg of cement is related to 0.84 kg CO_2_ eq. while for 1 kg of reinforcement, the result of the climate change indicator is 0.47 for steel and 2.01 kg CO_2_ eq. for glass, respectively. Finally, the findings from the comparison of slabs designed for the LBC and SA limit states were not expected. Although the relative decline of the modulus of elasticity was more significant than the relative decrease of compressive strength compared to the reference, a comparison of the environmental impacts based on the limit states between NAC and fRCAC, and fRMAC, is not essential. 

A comparison of the reinforcement type indicated a slightly better environmental performance for glass fibers in the case of the LBC limit state; however, the results for the SA limit state showed a completely opposite trend, where the environmental impact of glass fibers reinforcement is significantly higher. The probable reason is the increase in slab thickness correlated with a minimal percentage of glass fiber reinforcement. For this reason, the concrete volume increase leads to higher environmental impacts.

**Figure 4:**
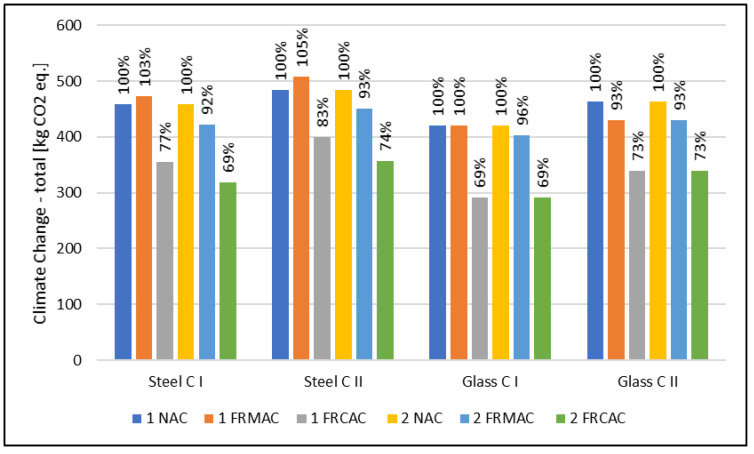
Results of climate change for concrete slab designed according to the load-bearing capacity limit state. (1) The compensation by varying thickness of slab; (2) the compensation by varying reinforcement level.

**Figure 5:**
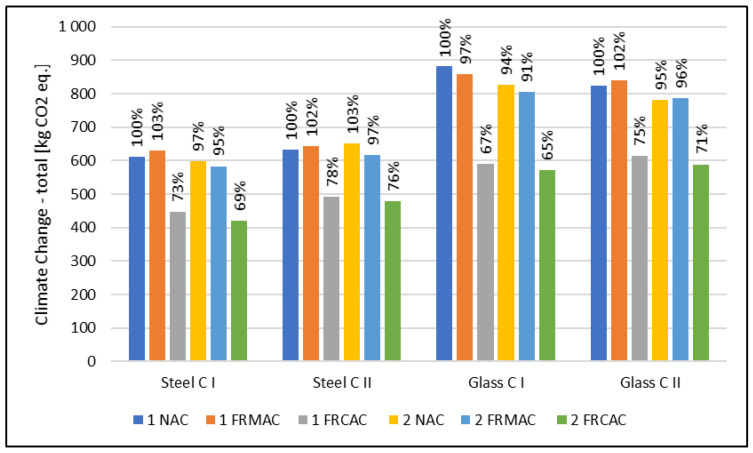
Results of climate change for concrete slab designed according to the serviceability limit state. (1) The compensation by varying thickness of slab; (2) the compensation by varying reinforcement level.

In the case of the climate change indicator describing the effect on global warming (see Figure 4 and Figure 5), the results of the environmental assessment show a positive influence of replacing natural sand with an fRCA. On the contrary, the use of an fRMA does not achieve favorable results where it is similar to the normalized and weighted results caused by the decrease of compressive strength, which is essential for a design based on the LBC limit state, and the modulus of elasticity is crucial for a design based on the SA limit state. The decline of mechanical properties is also essential, and the positive influence connected with replacing natural sand is negligible. The reduction of greenhouse gases (GHGs) related to mixtures with the full replacement of natural sand by an fRCA is between 84 kg CO_2_ eq and 141 kg CO_2_ eq for a design based on the LBC limit state and 140 kg CO_2_ eq and 308 kg CO_2_ eq for a design based on the SA limit state, which shows a decrease of 23% to 31% and 22% to 35%, respectively. In the case of an fRMA, a similar or slightly higher potential impact on the climate change indicator in comparison with a conventional solution (NAC) can be observed. The maximal increase was 23 kg CO_2_ eq which corresponds to 5%.

Generally, from the reinforcement type point of view, the results of the climate change indicator differ for limit states. In the case of the LBC limit state, a more sustainable option seems to be glass fiber reinforcement; on the contrary, in the case of the SA limit state, steel reinforcement can be observed as the more favorable solution. It is possible to observe similar trends for all assessed types of concrete slabs. For this reason, the type of reinforcement is not straightly correlated with the concrete type.

In the case of compensating the properties of an fRAC by the (1) varying thickness of the slab and (2) varying reinforcement level, the results are similar to normalized and weighted results. For all assessed mixtures, the reduction of the climate change impact is more significant when the decline of properties is compensated by more reinforcement.

In addition, concrete mixtures with two amounts of cement were assessed. Concrete (I) contained 260 kg/m^3^ of OPC and (II) with 300 kg/m^3^ of OPC. In the comparison, in the perspective of the design oriented to the load-bearing limit state, the result of the climate change indicator is slightly higher for concretes containing more cement where the design of the concrete slab is based on the LBC limit state, and so compressive strength. On the contrary, in the SA limit state point of view, the GWP differs significantly, and it is more favorable for mixtures with higher amounts of cement and, consequently, better mechanical properties, especially the modulus of elasticity.

**Figure 6:**
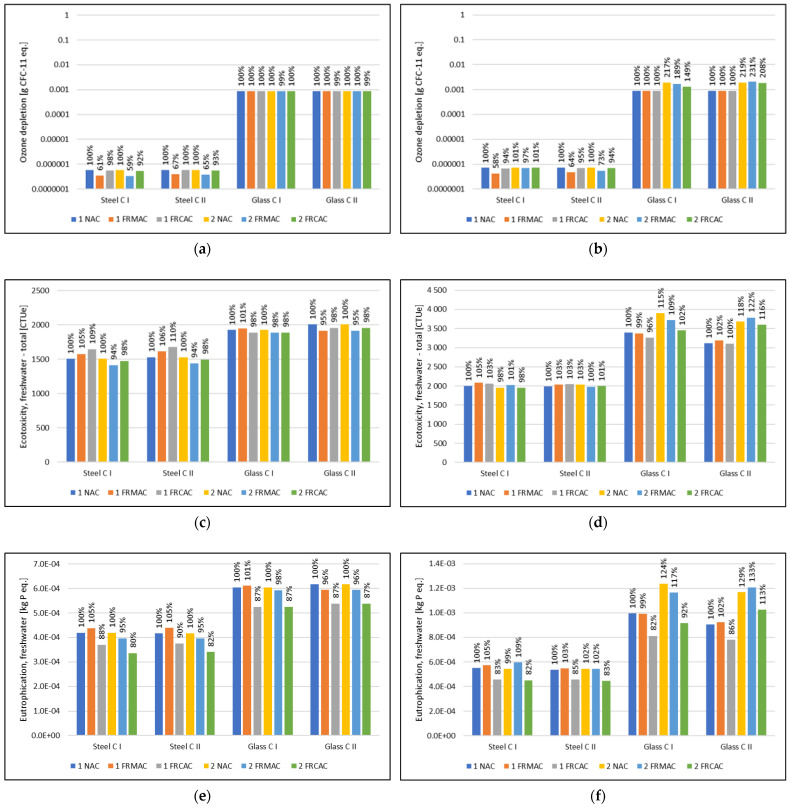

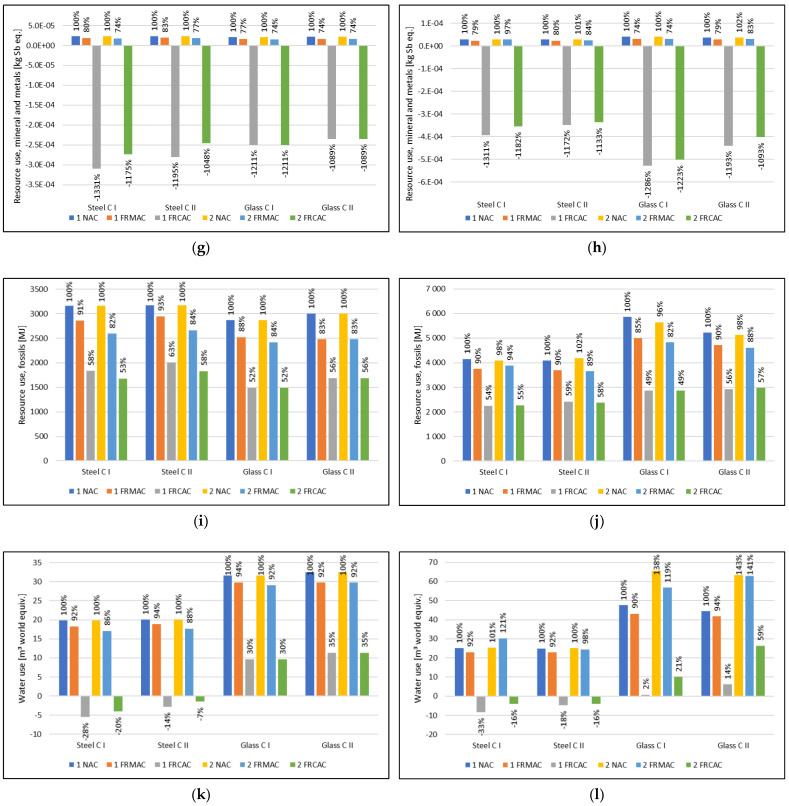
Results of selected impact indicators (characterization according to EF 3.0), concrete slab designed according to load-bearing capacity (**a**,**c**,**e**,**g**,**i**,**k**) and the serviceability limit state (**b**,**d**,**f**,**h**,**j**,**l**); design scenario using (1) the compensation by varying thickness of slab or (2) the compensation by varying reinforcement level; (**a**,**b**) ozone depletion (ODP), (**c**,**d**) ecotoxicity, freshwater total (ETP), (**e**,**f**) eutrophication, freshwater (EP), (**g**,**h**) resource use, mineral and metals (AD-min.), (**i**,**j**) resource use, fossils (ADP-fos.), (**k**,**l**) water use (WDP).

Considering the results of the climate change indicator, similar conclusions can be drawn based on the results of other environmental indicators, which are presented in the Supplementary Table S1. The aspects significantly contributing to particular indicators can be discussed based on Figure 6, which presents indicators representing a broader scale of potential environmental impact chains.

The result of the potential impact in the ozone depletion category is influenced by the use of glass reinforcement production (see Figure 6a,b), therefore ODP has higher results when glass roving is used.

The results of the ecotoxicity potential indicator are in accordance with results of the climate change indicator (see Figure 6c,d). The contribution of recycling concrete is negligible. On the other hand, this indicator is affected by transport processes (including diesel production) and the production of raw materials such as cement. 

In the case of eutrophication potential (Figure 6e,f), the production of epoxy resin used for glass reinforcement significantly contributes to the result of the EP indicator. Recycling steel scrap from construction and demolition waste, which is used for the production of recycled concrete aggregate, contributes beneficially to several indicators, not only those that are resource-related (see Figure 6g,h). The effect of these environmental benefits, which represent the prevention of environmental impacts in the case of steel recycling, can be observed in the results of ADP-min. and ADP-fos. The beneficial impact of steel scrap recycling was presented in a previous study [28]. While the benefit of this process leads to an overall negative value for results of ADP-min. related to mixtures in which RCA is used, in the perspective of ADP-fos. these benefits result in a rather small decrease of impacts (27–36% in comparison with fRMAC). The ADP-fos. indicator is also affected by the production of cement and fuel for transport (diesel). Another significantly contributing process is the landfilling of disposed concrete slabs, which can reach more than half of the cement production impact. Furthermore, in this indicator, the impact of steel scenarios designated for LBC is slightly higher than the results of slabs with glass reinforcement, which is in accordance with the results of the climate change indicator. 

The impact in water depletion potential mainly comes from cement production and landfilling, but an even higher impact is caused by epoxy resin production, which contributes more than the total impacts related to cement production and landfilling. Despite the burden of epoxy resin production, in the comparison of materials for roving, glass and steel reinforcement reach approximately the same result of WDP. 

Based on results presented in Figure 6, use of RCA contributes beneficially to several impact indicators. Moreover, there is a difference between impacts of scenarios with different reinforcement materials, but also production of epoxy resin effects the result of WDP and EP.

## 4. Conclusions

In this study, the environmental impact of concrete slabs made of recycled aggregate concrete where the natural sand is fully replaced by the fine recycled masonry aggregate and fine recycled concrete aggregate was analyzed. The design of the structural elements was established according to the experimentally verified properties of concrete. The design of the slab was developed aiming at two limit states (load-bearing and serviceability) and considering the use of two different reinforcement materials (steel and glass). Moreover, two approaches to compensate for the decrease in recycled aggregate properties were used to design 48 scenarios.

Based on the results of the environmental assessment of these scenarios using the LCA method, the following conclusions can be drawn:

- Generally, the results show a strong positive influence on the environmental impacts for the mixtures containing a fine recycled concrete aggregate. The decrease in the result of the climate change indicator was essential. However, the replacement of natural sand by fine recycled concrete aggregate has either no impact, or an insignificant impact, on the mechanical properties of concrete, which is the probable reason for very positive results in the LCA analysis.
- In the case of compensation for the decline of mechanical properties, the results show a lower environmental impact for a higher reinforcement level in comparison with increasing the slab thickness.
- The design of the slab was based on the limit states of load-bearing capacity (LBC) and serviceability (SA), where the LBC is based on the compressive strength and SA on the modulus of elasticity. The results of the LCA case study show the differences between these two approaches. Generally, the concrete slab was significantly larger in the SA design approach. In addition, the results of the environmental assessment differ in the case of reinforcement type, where the results for these two design approaches were exactly the opposite. The use of steel reinforcement is more advantageous for the SA limit state and, on the contrary, the glass fibers are better in the LBC approach, which is caused by the increase of slab thickness due to the minimal reinforcement level.
- The results of the climate change indicator show similar or slightly higher values for mixtures containing a fine recycled masonry aggregate than reference mixtures with natural sand. On the contrary, the normalized and weighted results show a slightly positive impact of replacing the natural sand, which shows a positive effect in the other impact categories.

This study presented a comprehensive approach to the LCA case study of the concrete structural elements with the full replacement of sand in the mixture. The results showed a clear positive environmental impact, especially for the fine recycled concrete aggregate, where the decline of mechanical properties was insignificant and so the environmental benefits are essential. According to the reported results, the use of a fine recycled aggregate as a substitution for natural sand in the concrete mixture has clear environmental benefits, which not only prevent the erosion and destruction of the landscape, but also lead to reduced global impacts such as climate change.

## Figures and Tables

**Table 1:** The physical properties of each fraction of aggregates used for concrete mixtures.

RA Types	Grading (mm)	Finest Particles Content	Oven-Dried Particle Density		Water Absorption Capacity	
		f (%)	ρRD (kg/m^3^)	σ	WA24 (%)	σ
Natural aggregate (NA1)	0–4	0.3	2570	81	1.0	0.0
	4–8	0.3	2530	12	1.7	0.3
	8–16	0.4	2540	12	1.9	0.2
Fine recycled masonry aggregate (fRMA)	0–4	1.0	2320	130	6.6	0.8
Fine recycled concrete aggregate (fRCA)	0–4	0.6	2430	60	3.6	0.8

**Table 2:** Concrete mix proportion, per cubic meter.

Concrete Mixture	Cement	Mixing Water + Additional Water	w/c Ratio	Natural Aggregate		Recycled Aggregate
				Fine	Coarse	Fine
	(kg/m^3^)	(kg/m^3^)	(-)	(kg/m^3^)	(kg/m^3^)	(kg/m^3^)
NAC I	260	169 + 0	0.65	709	1130	0
fRMAC I	260	169 + 18	0.72	0	766	971
fRCAC1 I	260	169 + 17	0.71	0	949	843
NAC II	300	165 + 0	0.55	671	1167	0
fRMAC II	300	165 + 17	0.61	0	822	920
fRCAC1 II	300	165 +16	0.60	0	994	800

**Table 3:** Average values and standard deviation of evaluation of density and mechanical properties of concrete at age of 28 days.

Recycled Concrete Mixture	Dry Density		Compressive Strength		Target Concrete Strength Class	Flexural Strength		Static Modulus of Elasticity	
Designation	(kg/m^3^)	(kg/m^3^)	(MPa)	σ	(-)	(MPa)	σ	(GPa)	σ
NAC IA	2301	2301	33.2	2.5	C20/25	6.2	0.2	36.7	1.4
fRMAC IA	2181	2181	30.0	2.2	C20/25	5.5	0.4	22.4	1.0
fRCAC1 IA	2276	2276	34.4	1.7	C25/30	5.8	0.3	29.6	0.4
NAC IIA	2324	2324	44.9	0.9	C30/37	7.6	0.9	35.9	0.5
fRMAC IIA	2191	2191	38.0	0.9	C25/30	6.8	0.6	25.3	0.2
fRCAC1 IIA	2278	2278	42.9	0.8	C30/37	6.5	0.4	31.4	1.0

**Table 4:** Average values and standard deviation of evaluation of the flexural strength before and after freezing–thawing the frost resistance coefficient and carbonation depth of concrete mixtures.

Recycled Concrete Mixture	Flexural Strength + Standard Deviation				Frost Resistance Coefficient	Freeze–Thaw Resistance	Carbonation Depth
Designation	0 Cycles		100 Cycles		(-)	Cycles	(mm)
NAC IA	6.15	±0.22	6.87	±0.20	1.12	100	2.78
fRMAC IA	5.53	±0.39	5.85	±0.40	1.06	100	7.10
fRCAC1 IA	5.78	±0.30	6.57	±0.26	1.14	100	4.51
NAC IIA	7.55	±0.87	7.80	±0.12	1.03	100	0.77
fRMAC IIA	6.84	±0.60	6.78	±0.00	0.99	100	1.71
fRCAC1 IIA	6.54	±0.44	6.73	±0.10	1.03	100	0.57

**Table 5:** Limit state of load-bearing capacity, results of design and calculations.

Design	Mixture	t	d_s_	a	c	M_Ed_	M_Rd_	V_conc._	W_reinf._
	Designation	(mm)	(mm)	(mm)	(mm)	(kNm)	(kNm)	(m^3^)	(kg)
Steel reinfor., thickness modification	NAC IA	230	10	100	20	65.593	66.532	1.380	36.992
	fRMAC IA	260	10	100	35	70.149	71.149	1.560	36.992
	fRCAC1 IA	260	10	100	35	70.149	71.812	1.560	36.992
	NAC IIA	220	10	100	20	64.074	64.218	1.320	36.992
	fRMAC IIA	250	10	100	35	68.63	68.795	1.500	36.992
	fRCAC1 IIA	250	10	100	35	68.63	69.204	1.500	36.992
GFRP reinf., thickness modification	NAC IA	210	6	75	15	62.555	62.562	1.260	4.750
	fRMAC IA	230	6	75	15	65.593	68.933	1.380	4.750
	fRCAC1 IA	210	6	75	15	62.555	62.722	1.260	4.750
	NAC IIA	210	6	75	15	62.555	63.68	1.260	4.750
	fRMAC IIA	210	6	75	15	62.555	63.126	1.260	4.750
	fRCAC1 IIA	210	6	75	15	62.555	63.542	1.260	4.750
Steel reinfor., reinforcem. Modification	NAC IA	230	10	100	20	65.593	66.532	1.380	36.992
	fRMAC IA	230	10	92	35	65.593	65.826	1.380	40.209
	fRCAC1 IA	230	10	93	35	65.593	65.934	1.380	39.776
	NAC IIA	220	10	100	20	64.074	64.218	1.320	36.992
	fRMAC IIA	220	10	90	35	64.074	64.696	1.320	41.102
	fRCAC1 IIA	220	10	91	35	64.074	64.519	1.320	40.651
GFRP reinf., reinforcem. modification	NAC IA	210	6	75	15	62.555	62.562	1.260	4.750
	fRMAC IA	220	6	76	15	64.074	64.682	1.320	4.688
	fRCAC1 IA	210	6	75	15	62.555	62.722	1.260	4.750
	NAC IIA	210	6	75	15	62.555	63.68	1.260	4.750
	fRMAC IIA	210	6	75	15	62.555	63.126	1.260	4.750
	fRCAC1 IIA	210	6	76	15	62.555	62.739	1.260	4.688

**Table 6:** Limit states of serviceability, results of design, and calculations.

Design	Mixture	t	d_s_	a	c	M_Ed_	M_Rd_	V_conc._	W_reinf._	s
	Designation	(mm)	(mm)	(mm)	(mm)	(kNm)	(kNm)	(m^3^)	(kg)	(mm)
Steel reinfor., thickness modification	NAC IA	310	10	100	20	77.743	93.85	1.860	36.992	23.98
	fRMAC IA	350	10	100	35	83.818	101.882	2.100	36.992	23.29
	fRCAC1 IA	330	10	100	35	80.78	95.715	1.980	36.992	22.84
	NAC IIA	290	10	100	20	74.705	88.121	1.740	36.992	22.66
	fRMAC IIA	320	10	100	35	79.262	92.698	1.920	36.992	23.75
	fRCAC1 IIA	310	10	100	35	77.743	89.693	1.860	36.992	22.01
GFRP reinf., thickness modification	NAC IA	450	6	75	15	99.005	145.172	2.700	4.750	21.34
	fRMAC IA	480	6	75	15	103.562	154.985	2.880	4.750	21.81
	fRCAC1 IA	440	6	75	15	97.487	141.89	2.640	4.750	22.23
	NAC IIA	380	6	75	15	88.374	122.195	2.280	4.750	20.99
	fRMAC IIA	420	6	75	15	94.449	135.41	2.520	4.750	20.4
	fRCAC1 IIA	390	6	75	15	89.893	125.499	2.340	4.750	21.15
Steel reinfor., reinforcem. Modification	NAC IA	300	12	120	20	76.224	107.28	1.800	44.391	23.88
	fRMAC IA	300	12	45	35	76.224	242.309	1.800	118.375	23.98
	fRCAC1 IA	300	12	85	35	76.224	140.324	1.800	62.669	23.64
	NAC IIA	300	12	170	20	76.224	77.554	1.800	31.335	22.35
	fRMAC IIA	300	12	90	35	76.224	134.045	1.800	59.188	23.71
	fRCAC1 IIA	300	12	135	35	76.224	91.487	1.800	39.458	23.88
GFRP reinf., reinforcem. modification	NAC IA	410	10	96	15	92.93	274.715	2.460	10.308	21.74
	fRMAC IA	440	10	110	15	97.487	259.314	2.640	8.996	23.34
	fRCAC1 IA	420	10	140	15	94.449	197.432	2.520	7.069	23.42
	NAC IIA	350	10	95	15	83.818	237.517	2.100	10.417	23.27
	fRMAC IIA	380	10	90	15	88.374	270.969	2.280	10.996	23.16
	fRCAC1 IIA	360	10	100	15	85.337	232.763	2.160	9.896	23.67

**Table 7:** Normalized and weighted results (EF 3.0) and differences −Δ (%) of limit state of load-bearing capacity (LB).

Scenario	EF 3.0.	−Δ (%)	Scenario	EF 3.0.	−Δ (%)	Scenario	EF 3.0.	−Δ (%)	Scenario	EF 3.0.	−Δ (%)
1S NAC I	0.024	0.0	1S NAC II	0.025	0.0	1G NAC I	0.022	0.0	1G NAC II	0.024	0.0
1S fRMAC I	0.023	1.4	1S fRMAC II	0.025	−0.7	1G fRMAC I	0.021	4.2	1G fRMAC II	0.021	10.6
1S fRCAC I	0.018	26.0	1S fRCAC II	0.020	21.0	1G FRCAC I	0.015	33.2	1G fRCAC II	0.017	29.4
2S NAC I	0.024	0.0	2S NAC II	0.025	0.0	2G NAC I	0.022	0.0	2G NAC II	0.024	0.0
2S fRMAC I	0.021	11.6	2S fRMAC II	0.022	10.2	2G fRMAC I	0.020	8.1	2G fRMAC II	0.021	10.6
2S fRCAC I	0.016	33.4	2S fRCAC II	0.017	29.3	2G fRCAC I	0.015	33.2	2G fRCAC II	0.017	29.4

**Table 8:** Normalized and weighted results (EF 3.0) and differences −Δ (%) of serviceability (SA).

Scenario	EF 3.0.	−Δ (%)	Scenario	EF 3.0.	−Δ (%)	Scenario	EF 3.0.	−Δ (%)	Scenario	EF 3.0.	−Δ (%)
1S NAC I	0.032	0.0	1S NAC II	0.032	0.0	1G NAC I	0.046	0.0	1G NAC II	0.042	0.0
1S fRMAC I	0.031	1.5	1S fRMAC II	0.031	2.1	1G fRMAC I	0.042	6.8	1G fRMAC II	0.041	2.0
1S fRCAC I	0.022	30.4	1S fRCAC II	0.024	25.6	1G fRCAC I	0.029	35.9	1G fRCAC II	0.030	28.5
2S NAC I	0.031	2.3	2S NAC II	0.033	−2.7	2G NAC I	0.044	4.4	2G NAC II	0.041	3.0
2S fRMAC I	0.030	5.9	2S fRMAC II	0.030	5.5	2G fRMAC I	0.040	11.1	2G fRMAC II	0.039	5.9
2S fRCAC I	0.021	33.4	2S fRCAC II	0.023	27.6	2G fRCAC I	0.029	37.0	2G fRCAC II	0.030	29.6

## Data Availability

Not applicable.

## Supplementary Materials

The following supporting information can be downloaded at: https://www.mdpi.com/article/10.3390/ma16020616/s1. Table S1: Results of environmental indicators according to the EF 3.0 characterization method.
